# Face Masks Are Beneficial Regardless of the Level of Infection in the Fight Against COVID-19

**DOI:** 10.1017/dmp.2020.320

**Published:** 2020-09-09

**Authors:** Mervin L. Burnett, Consolato M. Sergi

**Affiliations:** Department of Laboratory Medicine and Pathology, Stollery Children’s Hospital, University of Alberta, Edmonton, AB, Canada

**Keywords:** competency, COVID-19, droplets, facemasks, public health

## Abstract

Coronavirus disease 2019 (COVID-19) due to severe acute respiratory syndrome coronavirus 2 (SARS-CoV-2) is currently a global pandemic that has affected over 7 million people worldwide, resulting in over 400,000 deaths. In the past 20 years, they have been several viral epidemics that were primarily transmitted by respiratory droplets. The use of face masks is proven to be effective in protecting health-care workers as they perform their duties. Still, there is limited evidence about whether the widespread use of face mask would be very useful in protecting the general population. This study aimed to conduct a review to determine if face masks would be beneficial in the general population as a means of reducing the spread of COVID-19. The widespread implementation of wearing face masks by the general population is challenging due to a variety of factors. However, the extensive use of cloth masks in conjunction with other preventative measures such as social distancing and handwashing can potentially reduce the risk of transmission of COVID-19.

The World Health Organization (WHO) reports that viral diseases continue to emerge and represent a severe threat to public health.^1^ Over the past 20 years, there have been several viral epidemics, including severe acute respiratory syndrome - coronavirus (SARS-CoV) in 2002 to 2003, H1N1 influenza in 2009, and Middle East respiratory syndrome coronavirus (MERS-CoV) in 2012.^1,2^ In December 2019, an unknown disease characterized by pneumonia occurred in Wuhan, Hubei Province, China. On January 7, 2020, a novel coronavirus (severe acute respiratory syndrome coronavirus 2, SARS-CoV-2) was identified as the causative agent for the cluster of respiratory illnesses (coronavirus disease 2019, or COVID-19), which were present in Wuhan.^3-6^ A common sight is images of people wearing masks in public. The use of face masks has been prevalent in Asian countries, especially China, South Korea, and Japan, since the outbreak of COVID-19.^7,8^ Face masks have been commonly seen, especially in Asian countries, during the outbreaks of other viral epidemics. In China, some provinces and municipalities have enforced compulsory mask-wearing in public. However, China’s national guidelines have recommended a risk-based approach for mask-wearing among health-care workers and the general public.^7^

The current outbreak dynamics of COVID-19 strongly indicate human to human transmission, which triggered a humanitarian and economic disaster to multiple countries.^1^ Currently, there is no clinical approved antiviral drug or vaccine for COVID-19.^1,2^ Therefore, it is crucial that measures be implemented to decrease the spread of this disease from person to person. The WHO, which has been a pillar in effectively tackling several epidemics in the past, currently recommends social distancing, social isolation, and regular hand washing or hand sanitization using an alcohol-based rub.^9^ The wearing of face masks by the general population has not been recommended by the WHO until June 5, 2020.^10^ Recently, the Centers for Disease Control and Prevention (CDC) has advised that persons should wear cloth masks in situations where social distancing would be challenging to maintain.^11^ It might be time to rethink the recommendation of wearing masks, as the spread of COVID-19 is rapidly increasing, and the death toll is rising particularly in vulnerable layers of the society.

## Transmission

The primary source of infection remains unknown, but human to human transmission was observed early after the emergence of COVID-19. Genomic analysis of SARS-CoV-2 showed a phylogenetical relationship to several severe acute respiratory syndrome-like (SARS-like) viruses. These data suggest that bats could be the possible primary reservoir, while the intermediate host and method of transfer to humans remain unknown. The transmission of COVID-19 is human to human, and symptomatic people are the most frequent source of spread. The most common symptoms at the onset of COVID-19 are fever, cough, and fatigue.^2,3^ Other symptoms include sputum production, headache, hemoptysis, diarrhea, dyspnea, and lymphedema.^2,3^ In severe cases, COVID-19 can progress to pneumonia, multi-organ failure, and death. On average, 97.5% of people will develop symptoms within 12.5 days of infection.^1^

Data suggest that presymptomatic and asymptomatic individuals can also spread the virus. Like other respiratory pathogens, the transmission of COVID-19 occurs through aerosol droplets from coughing and sneezing.^1,2^ The incubation period for the virus is generally within 3 to 7 days but could be as long as 14 days.^6^ Also, the R_0_ seems now to be quite different from what it has been calculated originally (2.2-2.7). The primary reproductive number, R_0_, relies on the exponential growth rate of an outbreak, as well as additional factors, such as the latent period and the infectious period. Most recently, Sanche et al. found R_0_ is likely to be 5.7 with a broad 95% CI (3.8-8.9).^12^ This value may better explain the pandemic spread than a R_0_ of approximately 2.5.

## Preventative Measures

Preventative measures in the community are essential to stopping the spread of COVID-19. The most effective preventive measures are washing hands with soap and water. Also, the preventive measures include the use of an alcohol-based hand sanitizer, avoidance of touching eyes, nose, or mouth, proper coughing or sneezing into a tissue or a bent elbow, wearing a mask with respiratory symptoms, and maintaining social distancing of at least 6 feet (2 meters).^9^ The initial recommendation by the WHO was that only people who are sick and show symptoms and persons who are caring for people who are suspected of having COVID-19 should wear masks.^9^ However, the recommendations about the use of a face mask for the public varies among countries. Masks used by the general public include cloth masks, dust masks, surgical masks, and N95 masks. In reviewing the chronologic deployment of the COVID-19 pandemic, there seems to be a certain degree of ambiguities in statements. In economics, uncertainty, and a decision-maker response to it, can be modeled formally in the context of a general decision model.^13^ Both ambiguity and ambiguity attitudes may harbor a very detrimental effect on several countries with delays of prompt action. The most recent recommendations by the CDC is that cloth face coverings should be used in public settings where social distancing measures are challenging to maintain.^11^ The CDC advises that the use of cloth face coverings would slow rather than stop the transmission of the virus as they are not as effective as surgical masks or N95 masks. Surgical masks and N95 masks are considered critical supplies, and the CDC advises that they should be reserved for use by health-care workers who are at a higher risk.

## DISCUSSION

Face masks combined with other preventative measures, such as social distancing and regular hand washing, can play a crucial role in reducing the spread of COVID-19.^7,14^ Wearing a face mask in public may impede the spread of infectious diseases, including COVID-19, by preventing the inhalation, as well as, the exhalation and dissemination of infectious droplets.^7^ While it is established that COVID-19 is carried by airborne droplets, mostly from coughing and sneezing, there is some dispute as to the distance that these airborne droplets can travel. Researchers at Massachusetts Institute of Technology (MIT) in Cambridge, Massachusetts, recently experimented with laboratory conditions, which showed that coughs could project liquid up to 6 meters away. At the same time, sneezes, which involve higher velocities, can allow fluids to travel up to 8 meters.^15,16^ However, the current advised social distance is 2 meters. There have been early reports that COVID-19 is spreading rapidly between people, while most scientists agree that the virus is transmitted. Droplets, either directly or indirectly, on objects are of concern, and there is preliminary evidence that airborne transmission may indeed be occurring.^15^ Some experts suggest that the virus can be spread by either talking or breathing and can linger in the air and on surfaces hours after being expelled.^15^ Also, wearing face masks in public can prevent asymptomatic and presymptomatic persons from unknowingly transmitting the virus.^14^ As tests are only being conducted on people who present with symptoms or persons who are high risk, the number of COVID-19 cases do not include asymptomatic carriers, which could be spreading the virus unknowingly. Asymptomatic and presymptomatic infected persons are less likely to practice social isolation. There are substantial considerations that using facemasks provides an extra layer of protection.

Even though there are advantages to the general population wearing face masks, there are also disadvantages, such as persons who may become complacent and not adhere to the other recommendations, such as hand washing and social distancing. Persons may also hoard N95 respirators, which would increase prices and/or decrease the number of masks available for health-care workers, caregivers, and persons who are diagnosed with COVID-19.

Bin-Reza et al. conducted a systemic review published in 2011 that was conducted between November 2009 and January 2011.^17^ This meta-analysis included 17 studies, which included published randomized control trials, quasi-experimental, and observational studies.^17^ This meta-analysis was unable to establish a conclusive relationship between the use of face masks and protection against influenza or other respiratory infections.^17^ However, Jefferson et al. conducted a systemic review that investigated how physical barriers, including the use of face masks, would interrupt or reduce the spread of respiratory viruses.^18^ The meta-analysis of 6 case-control studies investigated by Jefferson et al. showed that surgical masks and N95 masks were highly effective in preventing the spread of SARS-CoV-1. The intervention effectiveness (odd ratio-1) for frequent handwashing (>10 times daily), wearing a mask, wearing an N95 mask, wearing gloves, wearing a gown, and handwashing, mask, gloves, and gown combined was 55, 68, 91, 57, 77, and 91%, respectively.^18^ The study concluded that, while the implementation of physical barriers could be difficult, these barriers, including face masks, can be useful in reducing the spread of respiratory infections in the general population.^19^

Davies et al. conducted a study that examined homemade masks as an alternative to commercial face masks. In this study, the researchers used 21 healthy volunteers to compare the efficacy of homemade masks made from cotton, surgical masks, and no masks on their capacity to block bacterial and viral aerosols.^20^ Results showed that surgical masks were 3 times more effective in preventing microorganisms than homemade masks and that 100% of the cotton was the best material to use for homemade masks. The study concluded that homemade cloth masks should only be considered as a last resort to prevent droplet transmission from infected individuals. Still, homemade cloth masks were better than no protection. Davies et. al recommended the use of medical masks rather than homemade cloth masks as a method of reducing transmission of infection from aerosols.^20^

There are potential challenges to the implementation of widespread face masks in the general population. Unless this order is mandatory, persons would have a choice whether to adhere to this recommendation. Sim et al. stated that individuals are more likely to wear facemasks due to the perceived susceptibility and perceived severity of the disease.^21^ Taylor et al. found that younger people are less willing to comply with wearing face masks than older persons, and that married people are more willing to wear face masks than persons who were never married.^22^ Several studies indicated that women were more likely to wear facemasks than men. At the same time, some cultures and ethnic groups, such as Asians, are more likely to wear facemasks than others.^22^ While face masks would play a critical role in reducing the spread of COVID-19, unless a mandatory order is in place, compliance would be a key influence on the overall outcome. Discordant and ineffective data have also been published and lead confusion to health-care administrators. Bae et al. studied the effectiveness of surgical and cotton masks in 4 patients.^23^ This article is flawed and has been retracted, but has generated false reports.^24^ The authors did not fully recognize the concept of limit of detection (LOD) of the in-house reverse transcriptase-polymerase chain reaction used in the study (2.63 log copies/mL), failing to express the values below LOD as “<LOD (value).” Numerous comments were added to the original publication in *Annals of Internal Medicine*. Inexplicably, the researchers who published in this journal discovered a 36-fold reduction to be “ineffective.” We and others disagree, and the editors decided for a retraction of the article.^24^

While there is a debate about the evidence that face masks can provide adequate protection in the broad community, face masks are widely used in medical settings by health-care professionals during a wide range of medical procedures and practices, including as a precaution when providing care to patients with respiratory infections. As both N95 and surgical masks are used by health-care personnel for protection against airborne diseases such as SARS-CoV-1 and the influenza virus, it is reasonable to think that these masks would protect the general population worldwide. However, the public would need to be trained about the correct way of putting on, wearing, removing, and disposing of face masks for this to be effective. Social media may be useful in obtaining the level of knowledge for the general population. Face masks would have to be used in conjunction with other preventative measures such as social distancing and regular hand washing for it to be effective. However, as cloth masks can be used as a last resort, the use of cloth masks in the general population would provide an extra layer of protection while conserving the stocks of N95 and surgical masks for health-care workers and persons diagnosed with COVID-19.

### Recommendations for Further Research

There is a need for further research about the effectiveness of the widespread use of face masks by the general population comparing Asian and non-Asian “ethnicities.” Furthermore, research on the duration of protection for face masks, measures to prolong the life of disposable face masks, and the possible invention of reusable face masks should be encouraged. Leadership/management is a specialty that requires proficiency and executives should be considered by careful evaluation of the following 5 categories: (1) communication and relationship management, (2) knowledge of the health-care environment, (3) professionalism, (4) business skills and principles, and (5) leadership. A peer review of some actions during this pandemic should be undertaken by an interdisciplinary and independent group of stakeholders interested in improving world health-care leadership. Remarkably, any face-worn devices are used as personal protection equipment may be considered a crucial and compelling technology against epidemics if seen under the light of a historical, anthropological approach of their invention during the 1910-11 Manchurian plague outbreak.^25^

### Limitations

The major limitation of this study was the scarcity of publications that focused on the use of face masks by the general public for the prevention of the transmission of COVID-19. Also, there was a lack of studies that looked at the use of face masks in the general population to prevent the spread of respiratory diseases; rather studies focused on prevention in health-care workers and hospital settings.

## CONCLUSIONS

There is not enough scientific evidence to justify whether the wearing of face masks in the general population would reduce the spread of COVID-19. However, time is not on our side as COVID-19 is spreading rapidly through various parts of the world. The wearing of face masks or cloths combined with previous recommendations such as regular handwashing and social distance can further reduce the spread of COVID-19, as a protective mask may reduce the likelihood of infection, but not eliminate the risk for the transmission of COVID-19. While there is inconclusive evidence that the widespread use of face masks or cloth face coverings by the general population would decrease the spread of COVID-19, it should be considered given the seriousness of the current pandemic.
